# Multivariate Optimization of Tenax TA-Thermal Extraction for Determining Gaseous Phase Organophosphate Esters in Air Samples

**DOI:** 10.1038/s41598-019-40119-2

**Published:** 2019-03-04

**Authors:** Julius Matsiko, Honghua Li, Pu Wang, Huizhong Sun, Shucheng Zheng, Dou Wang, Weiwei Zhang, Yanfen Hao, Peijie Zuo, Yingming Li, Qinghua Zhang, Jianqing Zhang, Guibin Jiang

**Affiliations:** 1 State Key Laboratory of Environmental Chemistry and Ecotoxicology, Research Center for Eco-Environmental Sciences, Chinese Academy of Sciences, Beijing, 100085 China; 2 University of Chinese Academy of Sciences, Beijing, 100049 China; 3 Institute of Environment and Health, Jianghan University, Wuhan, 430056 China; 4 Shenzhen Center for Disease Control and Prevention, Shenzhen, 518055 China

## Abstract

Suitable conditions for thermal extraction of semi-volatile organic compounds have largely been arrived at by univariate optimization or based on the recommendations provided by the manufacturers of the extraction equipment. Herein, we demonstrated the multivariate optimization of Tenax TA–thermal extraction for determining organophosphate esters in the gas phase fraction of air samples. Screening and refining experiments were performed using the eighth fraction factorial and Box-Behnken designs, respectively, and satisfactory models were obtained. Subsequently, the process was optimized by Derringer’s desirability function and the global desirability was 0.7299. Following optimization, the analytes were desorbed at 290 °C for 10 minutes at a helium flow of 95 mL min^−1^, with the transfer line set at 290 °C. The analytes were then cryofocused at 20 °C and then cryodesorbed into the chromatographic column at 295 °C for 6 minutes. Method validation exhibited high linearity coefficients (>0.99), good precision (CV < 14%) and low detection limits (0.1–0.5 ng m^−3^). The method was tested by pumping 0.024 m^3^ of real indoor environment air through Tenax TA sorbent tubes. Furthermore, with multivariate optimization, analysis time and other resources were significantly reduced, and information about experimental factor interaction effects was investigated, as compared to the univariate optimization and other traditional methods.

## Introduction

Flame retardancy is certainly not a new phenomenon because its history can be traced back to about 2 centuries ago, when the Egyptians would soak and dry paper and wood to render these materials fire proof. Since then, different chemicals have been produced and used as additives or plasticizers in different consumer products to fulfill fire safety standards. However, most of these chemicals, such as polychlorinated biphenyls (PCBs), polybrominated diphenyl ethers (PBDEs), etc., were proved to be detrimental to both human and environmental health, and as a consequence, their production and usage were banned^1^. Placing a ban on the aforementioned chemicals didn’t mean an end to the risks associated with fire, but rather, the need to seek alternatives, which include organophosphate esters (OPEs)^2^.

OPEs are derivatives of phosphoric acid in which the three hydrogens of the acid have been replaced by alkyl-, aryl- or chloroalkyl-groups, e.g., triethyl phosphate (TEP), triphenyl phosphate (TPHP), tris (chloroethyl) phosphate (TCEP), etc. Due to their widespread production and usage, they have been found present in different environmental matrices, and like their predecessors (the legacy brominated flame retardants (BFRs)), OPEs have also been associated with harmful effects on both human and environmental health^3^. The occurrence and distribution of airborne OPEs are usually monitored by active or passive air sampling techniques and to that end, different active and passive sampler configurations, and their benefits and limitations, have been comprehensively discussed in the literature. Succeeding sampling, are the sample extraction (sonication, soxhlet, microwave assisted, accelerated solvent, elution) and cleanup (column chromatography ~silica, alumina, florisil and filtration ~glasswool, cotton wool) procedures, prior to instrumental analysis (gas/liquid chromatography-mass spectrometry)^4^. It is worth mentioning that large volumes of solvents and a lot of time, are involved in the sample extraction procedures that were discussed in the preceding citation. Nevertheless, thermal extraction (TE) techniques, which are rapid and solventless, have been reported^4–8^.

A plenitude of TE–gas chromatography/mass spectrometry (TE-GC/MS) applications in the analysis of semi-volatile organic compounds (SVOCs) exist in the literature. Additionally, remarkable reviews about the different TE setups, compatible sorbents, and pros and cons associated with this analytical technique, have been published in the past four decades. It can be recognized that TE procedures are influenced by various factors and as a consequence, suitable conditions necessary for the global analyte transfer from the sorbent to the chromatographic column need to be selected or optimized^9–12^. The univariate approach or recommendations by the thermal desorption equipment’s manufacturers (for some extraction parameters) have been utilized to select optimal conditions for TE procedures in most studies that have been developed to monitor SVOCs in different environmental media^5–8,13–19^. However, the univariate approach involves many analyses, especially when the experimental factors to optimize are many, and obviously, it consumes a lot of time and other resources. Besides, this approach also examines a limited experimental domain^20^, i.e., it does not provide information about the contribution of the interactions between the experimental factors to the values of the response factors. Actually, in some previous studies^5–8^, optimization of thermal extraction involving at least one OPE was demonstrated, but no information about the contribution of experimental factors’ interactions to the magnitude of the response factors was given. In addition, in our previous study^8^, there was also a need to calibrate the passive samplers against a high volume – active sampler for the calculation of sampling rates, which in itself is a limitation of passive air sampling (PAS). To address the limitations associated with the univariate optimization approach, chemometric tools, for example, multivariate modelling, coupled with global optimization, can be used. As a matter of fact, in some, but scarce studies, the multivariate approach has been used to optimize TE procedures to determine different SVOCs in different environmental media^21–23^. It is worth mentioning that the multivariate optimization approach has been utilized mainly for water and solid samples^21–24^, leaving a gap for application in air samples^24^. Based on the above limitation, we occasioned this study to fill the gap and pave the way for further studies of the same kind to involve other airborne semi-volatile organic compounds. Additionally, we thought that addressing some of the limitations that were encountered in the previously cited studies^5–8,13–19^, could be interesting.

We, therefore, attempted to utilize the multivariate approach to optimize a TE procedure based on Tenax TA as the sorbent, for the analysis of 10 OPEs in the gaseous phase fraction of air samples. Specifically, this study was guided by the following objectives: (1) Designing a series experiments to screen for the TE factors that significantly affect the response factors (compound specific chromatographic peak areas). (2) Utilizing response surface methodology (RSM) based on the Box-Behnken design (BBD) to develop second order polynomial models for evaluating the response factors in terms of TE factors that affect the response factors significantly. (3) Optimizing the best TE conditions for the target OPEs based on Derringer’s desirability function. (4) Validating the optimized procedure and applying it to real indoor air sampling of OPEs.

## Results

### Screening factorial design

The investigated factors and their ranges have been given in the methods section (Screening design). The design matrix together with the response factors’ results are presented in Table S1. The runs at the center were repeated twice (six replicates in total) in this case to ensure that the repeatability was within the normal dispersion range, and in terms of the coefficient of variation (CV), repeatability ranged from 4.0% for TCEP to 16.6% for EHDPP. Analysis of variance (ANOVA) results of the screening design experiments are presented in Table 1 in form of the relative significance (with their signs) of the main effects associated to each factor as well as the quadratic interaction of desorption flow. The positive sign means that the change of the experimental factor from the low level to high level increases the magnitude of the response factor, while a negative sign means that the change in the experimental factor from the low level to high level decreases the magnitude of the response factor. Desorption flow rate, TDS transfer line temperature, desorption hold time, desorption temperature and cryodesorption time had non-significant effects on the chromatographic peak areas of the target compounds. Cryofocusing temperature (E), cryodesorption temperature (F) and the quadratic effect of desorption flow rate (AA), had significant effects on the peak areas of at least one of the target compounds and their effects were therefore, evaluated by response surface methodology (RSM). The rest of the main experimental factors were not considered for optimization but were set at the central points when the BBD experiments were being performed.

**Table 1:** Experimental domain and the relative significance (with their sign) of the main effects associated to each factor as well as the quadratic interaction of desorption flow.

Experimental factors	Levels			Effects of the experimental factors on the compound specific chromatographic areas									
	(−1)	0	(+1)	TEP	TPP	TNBP	TCEP	TCIPP	TDCIPP	TBOEP	TPHP	EHDPP	TEHP
Desorption flow rate (A)	20	60	100	+	+	+	++	+	++	++	++	++	++
Desorption temperature (B)	260	290	320	+	+	−	+	−	++	++	++	++	++
TDS transfer line temperature (C)	260	290	320	++	+	+	+	+	++	+	++	++	++
Desorption time (D)	5	10	15	+	+	+	+	+	+	++	+	+	++
Cryofocusing temperature (E)	−100	−20	60	−−−	+	+++	+++	+++	+++	+++	+++	+++	+++
Cryodesorption temperature (F)	260	290	320	+++	++	++	++	++	++	++	++	++	+++
Cryodesorption time (G)	2	6	10	−	−	−	+	+	−	+	−	+	+
AA	20	60	100	−−−	−−−	−−−	−−−	−−−	−−−	−−	−−−	−−−	−−−

+++ or −−− indicate a statistically significant (*p* < 0.05) positive or negative effect. ++ or −−indicate a positive or negative effect that was close to statistical significance. + or − indicate a positive or negative effect that was far from reaching statistical significance.

### Response Surface Methodology (RSM)

**Figure 1:**
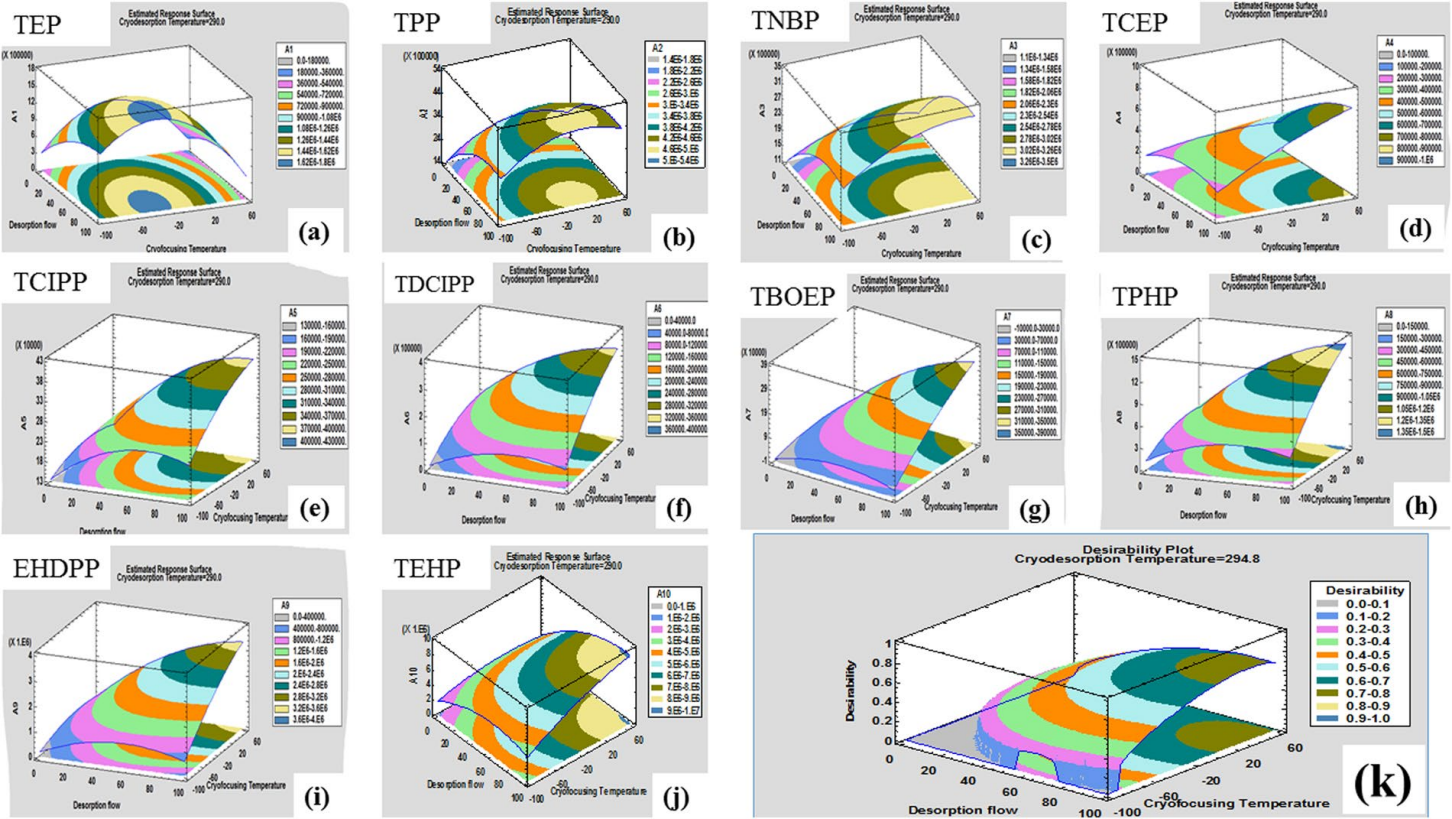
Response surface plots (**a**) to (**j**)) showing the effect of desorption flow (A) and cryofocusing temperature (B) at fixed cryodesorption temperature (C) on the peak areas of the target OPEs and on the global desirability (**k**). Cryodesorption temperature was fixed at the central point (290 °C) since it did not affect any of the responses significantly.

The letter codes for the studied factors in the BBD are A, B and C, respectively, for desorption flow rate, cryofocusing and cryodesorption temperatures. All experiments were performed in a randomized manner to minimize the bias effects of uncontrolled variables^25^. Three central points were incorporated to estimate the experimental error that is independent of the fitted model and to ensure that the variability was normally dispersed. The CVs of the results obtained from the central point experimental runs ranged from 6.8% for TEP to 19.6% for TPHP. The plot and experimental domain matrix for this design are shown in the Fig. S2. Additionally, the design matrix (with actual experimental factors’ values) together with the response factors’ results are presented in Table S2. The regression coefficients of the second order polynomial models (equation 6) expressing the relationship between the three experimental factors and response factors were computed based on the generated results and presented in Table 2. Analysis of variance (ANOVA) was used to evaluate the appropriateness of the model through the coefficients of determination as well as their corresponding adjusted values (Table 2). The statistical significance of the models’ regression coefficients was evaluated by the ANOVA *F*-test and the results are presented in Table 2. *P*–values less than 0.05 showed that the model terms had significant effects on the response factors. Meanwhile, the *F*-ratio and *p*- values for the lack of fit test ranged from 0.74 to 5.69 and from 0.1570 to 0.6720, respectively, indicating that the unexplained point cloud curvatures which could diminish the usefulness of the models do not exist in our design^26^. In other words, the models are adequate for the observed data at 95% confidence level. An example of ANOVA results for the fitted second order polynomial model of EHDPP is presented in Table S3. To understand the effect of the experimental factors on the response factors clearly, we generated the response surface plots of the models and illustrated them in a three dimensional space (Fig. 1(a–j)).

**Table 2:** The significance probability (*p* - value; *F*- ratio), lack of fit and coefficients of determination of second order polynomial models that demonstrated the relationship between the experimental and response factors as refined by the BBD.

OPE		Linear effects			Linear interactions			Quadratic interactions			Model	LOF	*r* ^2^	${r}_{adj}^{2}$
		A	B	C	AB	AC	BC	AA	BB	CC				
TEP	RC	−6578.31	−5362.24	68725.90	−41.71	67.82	−1.24	−86.11	−128.51	−123.33	−8.54E6		0.9090	0.7453
	*F*-ratio	2.51	7.41	0.18	1.08	0.40	0.00	1.07	37.96	0.69		5.69		
	*p*-value	0.1741	0.0417	0.6885	0.3457	0.5536	0.9823	0.3493	0.0016	0.4435	0.0368	0.1570		
TPP	RC	−20214.10	21629.90	192765.00	3.94	224.25	−71.02	−266.10	−127.83	−355.49	−2.32E7		0.7661	0.3450
	*F*-ratio	4.43	4.40	0.03	0.00	0.61	0.25	1.41	5.21	0.80		1.36		
	*p*-value	0.0893	0.0901	0.8646	0.9722	0.4699	0.6416	0.2882	0.0713	0.4129	0.2641	0.4840		
TNBP	RC	−7979.85	21520.60	131141.00	41.30	139.35	−70.44	−189.86	−54.09	−241.67	−1.59E7		0.7720	0.3615
	*F*-ratio	4.42	7.47	0.02	0.31	0.50	0.51	1.51	1.96	0.77		0.91		
	*p*-value	0.0895	0.0412	0.9000	0.6018	0.5127	0.5083	0.2737	0.2202	0.4191	0.2520	0.6070		
TCEP	RC	7444.20	4191.35	11763.40	30.20	1.54	−13.45	−40.86	−6.84	−20.55	−1.42E6		0.9404	0.8332
	*F*-ratio	13.48	53.65	0.06	6.93	0.00	0.77	2.93	1.31	0.23		2.00		
	*p*-value	0.0144	0.0007	0.8197	0.0463	0.9619	0.4194	0.1477	0.3037	0.6488	0.0139	0.3710		
TCIPP	RC	−23.90	3572.82	12392.20	7.90	13.15	−12.09	−21.58	−3.74	−23.40	−1.45E6		0.7846	0.3969
	*F*-ratio	4.83	8.45	0.06	0.89	0.35	1.17	1.53	0.73	0.57		0.74		
	*p*-value	0.0794	0.0335	0.8237	0.3896	0.5822	0.3291	0.2716	0.4310	0.4850	0.2264	0.6720		
TDCIPP	RC	2997.13	2853.14	12686.90	11.21	2.32	−8.71	−19.84	−4.52	−21.63	−1.77E6		0.9553	0.8747
	*F*-ratio	15.32	75.02	1.58	5.42	0.03	1.84	3.92	3.25	1.47		1.77		
	*p*-value	0.0113	0.0003	0.2645	0.0673	0.8636	0.2326	0.1046	0.1313	0.2789	0.0070	0.4040		
TBOEP	RC	5088.09	1724.69	8810.79	17.75	−4.76	−5.82	−17.27	−3.39	−13.68	−1.37E6		0.9724	0.9228
	*F*-ratio	30.49	114.02	5.20	18.73	0.19	1.13	4.10	2.52	0.81		3.46		
	*p*-value	0.0027	0.0001	0.0715	0.0075	0.6816	0.3360	0.0989	0.1730	0.4085	0.0022	0.2410		
TPHP	RC	11657.50	14648.60	71805.70	50.75	12.85	−47.80	−79.65	−16.74	−121.79	−1.02E7		0.9334	0.8134
	*F*-ratio	12.35	43.24	2.51	4.39	0.04	2.19	2.50	1.77	1.85		1.09		
	*p*-value	0.0170	0.0012	0.1740	0.0902	0.8500	0.1987	0.1748	0.2414	0.2321	0.0180	0.5520		
EHDPP	RC	45358.80	21252.60	135283.00	155.24	−12.61	−71.20	−224.65	−39.05	−229.32	−1.93E7		0.9714	0.9200
	*F*-ratio	28.33	110.25	1.02	16.13	0.01	1.91	7.79	3.77	2.57		1.87		
	*p*-value	0.0031	0.0001	0.3584	0.0102	0.9074	0.2256	0.0384	0.1099	0.1698	0.0024	0.3890		
TEHP	RC	12887.70	77284.80	417801.00	306.78	343.72	−261.83	−670.97	−113.61	−752.44	−5.49E7		0.9354	0.8193
	*F*-ratio	11.92	41.94	0.52	5.34	0.94	2.19	5.90	2.71	2.35		1.64		
	*p*-value	0.0182	0.0013	0.5017	0.0687	0.3760	0.1990	0.0595	0.1609	0.1860	0.0167	0.4260		

RC: Regression coefficient, A, B and C are the experimental factors, desorption flow, cryofocusing temperature and cryodesorption temperature, respectively. LOF refers to Lack of fit.

### Optimization process

For each of the responses, the partial desirability values were computed and are presented in Table 3, together with the global desirability that was obtained by optimizing the process simultaneously by searching from all design points and vertices. Under the aforementioned optimization considerations, the optimum conditions for the factors that were investigated in this design were as follows: desorption flow rate = 95.1069 mL min^−1^, cryofocusing temperature = 31.71 °C and cryodesorption temperature = 294.698 °C with a global desirability of 0.7299 (Table 3).

**Table 3:** Modelled and adopted optimum conditions for the significantly influential experimental factors, partial and global desirability of the peak areas, the predicted areas (and their 95% confidence limits) and the experimentally obtained peak areas.

Factor				Setting	Modified		
Desorption flow (A), mL/min				95.1069	95		
Cryofocusing Temperature (B), °C				31.71	20		
Cryo-desorption Temperature (C), °C				294.698	295		
**Response**	**Optimized**	**Prediction**	**Lower 95.0% Limit**	**Upper 95.0% Limit**	**Desirability**	**Validation (±SD, n = 3)**	**CV (%) of validated experimental values**
TEP	yes	1061000.0	648994.0	1474000.0	0.4786	978917.0 ± 112894.0	11.5
TPP	yes	4620000.0	3513000.0	5728000.0	0.8734	3702588.0 ± 334614.0	9.0
TNBP	yes	3243000.0	2480000.0	4007000.0	0.7477	3046976.0 ± 301440.0	9.9
TCEP	yes	739188.0	621161.0	857215.0	0.7102	595104.0 ± 49439.9	8.3
TCIPP	yes	362965.0	276627.0	449303.0	0.6574	326112.0 ± 21869.2	6.7
TDCIPP	yes	301971.0	252442.0	351500.0	0.5049	241696.0 ± 18558.9	7.7
TBOEP	yes	285892.0	243703.0	328081.0	0.5896	217899.0 ± 26699.6	12.3
TPHP	yes	1275000,0	1026000.0	1524000.0	0.8499	1107435.0 ± 90919.8	8.2
EHDPP	yes	3015000.0	2617000.0	3413000.0	0.8568	2295061.0 ± 260412.0	11.3
TEHP	yes	8525000.0	7159000.0	9890000.0	0.9383	6339173.0 ± 682202.0	10.8
Global desirability					0.7299		

### Chromatographic Separation

The compounds were completely separated under the chromatographic conditions that were used in our previous study^8^, and the results are presented in Table 4. Furthermore, the total ion chromatograms (TIC) for the field blank tube, intermediate calibration concentration and one of the real air samples are presented in Fig. S1.

**Table 4:** Target OPEs, retention time (*t*_*r*_), time window, monitoring ions (quantifier ions and qualifier, with their percentage abundances in brackets) used in SIM mode, regression analysis calibration data, accuracy, precision and instrumental detection and quantitation limits.

Targets	*t*_*r*_ min	Time window (min)	Monitoring ions		*r* ^2^	Regression equation	RRF, CV	Recovery (Repeatability, CV) (n = 5)			Intermediate precision, CV (n = 5)			LOD pg	LOQ pg
			m/z^a^	Q^b^				50 pg	500 pg	2000 pg	50 pg	500 pg	2000 pg		
TEP	7.05	6.54~9.46	99	155 (95), 127 (71)	0.9998	*Y* = 2.21*X*	2.9	117 (1.5)	90 (6.0)	96 (3.7)	4.4	5.0	8.1	2.7	9.0
TPP	11.90	9.46~13.64	99	141 (34), 183 (3)	0.9995	*Y* = 5.80*X*	4.7	127 (1.9)	103 (9.8)	107 (7.3)	4.9	3.6	7.7	3.7	12.0
TNBP	15.64	13.64~16.31	155	125 (27), 211 (25)	0.9989	*Y* = 4.34*X*	3.9	119 (6.5)	96 (8.5)	106 (7.4)	5.2	2.5	8.8	12.0	39.0
TCEP	17.15	16.31~20.15	249	251 (64)	0.9967	*Y* = 0.81*X*	9.6	107 (4.1)	91 (6.2)	111 (7.9)	7.4	4.5	8.1	6.6	22.0
TCIPP	17.46	16.31~20.15	277	279 (66)	0.9986	*Y* = 0.47*X*	5.6	127 (5.0)	104 (13.2)	108 (6.8)	4.5	3.4	7.4	9.5	32.0
TDCIPP	23.02	20.15~25.26	381	379 (62)	0.9975	*Y* = 0.28*X*	5.5	126 (4.4)	115 (2.2)	113 (2.0)	6.7	12.2	6.1	8.4	28.0
TBOEP	23.72	20.15~25.26	199	299 (39)	0.9969	*Y* = 0.20*X*	8.9	122 (6.7)	92 (9.5)	124 (9.8)	10.8	12.5	7.0	12.0	41.0
TPHP	23.83	20.15~25.26	326	325 (79)	0.9963	*Y* = 1.40*X*	6.1	133 (5.5)	109 (1.5)	102 (0.7)	4.5	11.7	3.2	11.0	37.0
EHDPP	24.00	20.15~25.26	251	250 (22)	0.9997	*Y* = 2.46*X*	8.9	82 (9.9)	93 (11.1)	121 (5.6)	11.6	3.8	10.6	12.0	44.0
TEHP	24.19	20.15~25.26	99	113 (25), 341 (16)	0.9942	*Y* = 6.95*X*	8.1	102 (5.2)	107 (8.9)	116 (3.8)	11.8	8.2	12.5	8.0	27.0

^a^Quantifier ion, ^b^qualifier ion (s).

### Method performance and validation

For purposes of calibration, five concentration levels were prepared from 50 to 2000 pg corresponding to 2.08 to 83.33 ng m^−3^ calculated for a sample volume of 0.024 m^3^ and experiments at each level, were performed in triplicate. Table 4 shows the results/values of statistical and analytical parameters obtained for each of the target OPEs.

### Application to real indoor air samples

We collected air samples from six different indoor environments (office (n = 3), bedroom (n = 2) and car (n = 1)). For all sampling campaigns, the ambient temperature was 23.2 ± 2.3 °C. As mentioned in the sampling section (under methods and materials), a QFF was used to seal the sampling end of the sorbent tube to prevent particles from entering the tube. Although some studies have indicated that some OPEs can get adsorbed on a QFF during sampling^27^, the sampling time of two hours was too short to influence the results significantly due the adsorption artifact^28^. The results are presented in Table 5. TPP, TNBP, TCEP, TCIPP, TPHP and TEHP were quantified in at least one of the sampled environments. Our results are in agreement with those obtained in some previous studies in which at least one of the target OPEs in this study were investigated in the gaseous fraction of air samples^5,29^.

**Table 5:** Detection and quantitation limits and real air sample results obtained from different environments.

	Concentrations (ng m^−3^)									
	TEP	TPP	TNBP	TCEP	TCIPP	TDCIPP	TBOEP	TPHP	EHDPP	TEHP
^a^MDL (ng m^−3^)	0.1	0.2	0.5	0.3	0.4	0.4	0.5	0.5	0.5	0.3
^b^mLOD (ng m^−3^)		0.2	0.5	0.5	0.08	nd	nd	0.02	nd	0.4
^a^MQL (ng m^−3^)	0.4	0.5	1.6	0.9	1.3	1.2	1.7	1.5	1.8	1.1
^b^mLOQ (ng m^−3^)		0.5	1.6	1.8	0.3	nd	nd	0.07	nd	1.2
Office 1	<0.4	<0.5	6.5	0.05	0.3	<1.2	<1.7	<0.07	<1.8	<1.2
Office 2	<0.4	2.2	<1.6	1.3	<0.3	<1.2	<1.7	<0.07	<1.8	<1.2
Office 3	<0.4	1.6	2.0	2.3	<0.3	<1.2	<1.7	<0.07	<1.8	<1.2
Bedroom 1	<0.4	<0.5	6.3	2.7	0.3	<1.2	<1.7	<0.07	<1.8	1.6
Bedroom 2	<0.4	<0.5	<1.6	6.2	0.4	<1.2	<1.7	<0.07	<1.8	3.7
Car	<0.4	<0.5	2.8	1.6	<0.3	<1.2	<1.7	0.1	<1.8	<1.2

^a^MDL and MQL are calculated by dividing the detection and quantification limits (LODs and LOQs) by the sampling volume.

^b^mLOD and mLOQ were calculated based on the signal: noise ratio of 3 and 10, respectively, for the compounds quantified in real air samples and then dividing by the sampling volume.

## Discussion

Full factorial design (FD) structures have benefits of efficiency, as well as allowing the experimenter to study the main factors and their interaction effects on process response factors. To allow the experimenter get information on the main effects and lower order interactions without having to run the full factorial design (FD), modification to fractional factorial designs (FFD) is performed. In factorial designs, each experimental factor is usually studied at two levels (low (−1) and high (+1)) although experimental studies at the central point (0) are also possible^25^. As a consequence, factorial designs are gaining interest in preliminary studies or in the initial steps of optimizing a given process^30,31^.

The fundamentals, advantages, limitations and applications of the response surface methodology (RSM) based on the BBD in optimizing chemical processes, were comprehensively reviewed by Ferreira *et al*.^24^. The analysis of variance (ANOVA) results (Table 2), showed that the models were appropriate with the coefficient of determination (*r*^2^) values ranging from 0.7661 for TNBP to 0.9724 for TBOEP. The adjusted coefficient of determination (${r}_{adj}^{2}$) values were also evaluated and are presented in Table 2. In general, the values of *r*^2^ and $\,{r}_{adj}^{2}$ indicated that the modelled response factors cover the point cloud of the experimental results satisfactorily. Besides, for each of the responses, the Durbin-Watson (DW) statistic had a *p*-value greater than 0.05, showing no indication of serial autocorrelation in the residuals at the 95% confidence level.

As depicted in Fig. 1(a), the peak area of TEP increased when desorption flow increased together with the simultaneous increase in cryofocusing temperature that was apparent below −20 °C. Thereafter, peak area reduced with increasing cryofocusing temperature and desorption flow as the linear interaction of desorption flow and cryofocusing temperature and quadratic interaction of cryofocusing temperature which have negative effects became important. The reason could be that the more volatile TEP is less effectively trapped at higher temperatures and flow rates. In Fig. 1(b), the simultaneous increase in desorption flow and cryofocusing temperature increases the peak area of TPP. However, after 20 °C, the interaction of the two has a negative effect on peak area although not significant. In Fig. 1c to j, the peak areas increase with the simultaneous increase in desorption flow and cryofocusing temperatures. Moreover, the interactions that are significant for example AB for TCEP, TBOEP and EHDPP, have positive effects on the peak areas. AA has a significant negative effect on the peak area of EHDPP as seen clearly in Fig. 1(j).

We observed that for all the compounds, except TEP (the most volatile), the peak areas generally increased with increasing cryofocusing temperature. Leon *et al*.^19^ attributed the same observation to the fact that, at lower cryofocusing temperatures, the transfer of the trapped analytes from the PTV injector to the chromatographic column may be ineffective due to frosting and water condensation or any other physical phenomena. Additionally, the peak areas were observed to increase with increase in desorption flow rate probably due to the adequate transfer of the analytes to the PTV injector. However, the peak areas are observed to decrease at higher desorption flow rates probably due to inadequate trapping of the analytes though for most of the compounds, the peak areas remained almost constant past the optimum desorption flow rate. It is worth noting that in RSM experiments, all variables are important, as there has been a preliminary screening to finding important variables, prior to exploring the surface^25^. Therefore, we never excluded any effects in the derivation of the second order polynomial models that give the relationship between the peak areas and the experimental factors.

Regarding optimization, the TE conditions would be considered as optimum if the peak area yields approached the maximum values. As a matter of fact, the goal of the process was to maximize the response factors. As seen in Fig. 1(a–j), the peak areas for different compounds are maximized by different conditions. Therefore, to obtain the optimal conditions (for the three factors evaluated) that are suitable for the extraction of all the compounds, we used the desirability function that was suggested by Derringer and Suich^32^, to simplify the response factor (peak area) for each compound. According to this function, each individual response “*Y*_*i*_” was transformed into a desirable dimensionless value, partial desirability (*d*_*i*_). The scale of the desirability function ranged from *d* = 0 for a completely undesirable response to *d* = 1 for a fully desired response. The 10 individual partial desirability functions were then weighted into a single composite response known as the global desirability function, D.

However, considering the operational parameters of the equipment, desorption flow and cryodesorption temperature were modified to 95 mL min^−1^ and 295 °C. Furthermore, by exploring the desirability change with decreasing cryofocusing temperature from the optimum setting of 31.71 °C, keeping the desorption flow at 95 mL/min (Fig. 1(k)), we adopted 20 °C as a compromised optimum temperature^25^ for trapping the analytes without affecting the value of the global desirability. Besides, at these conditions of desorption flow rate and cryofocusing temperature, it is clear from the response surface plots (Fig. 1(a to j) that the peak areas of the analytes remained within the desirable ranges. Additionally, at this temperature, the consumption of liquid nitrogen (coolant for the PTV) could be minimized^23^ since higher cryofocusing temperatures require a longer time for the CIS to equilibrate thereby consuming more liquid nitrogen prior to actual sample extraction. Cryofocusing at 20 °C has also been shown to produce good responses in previous studies^19,23^ in which the TE of SVOCs from water samples was investigated.

Under the modified optimal conditions for desorption flow, cryofocusing temperature and cryodesorption temperature, we fixed the insignificantly influential experimental factors (desorption temperature, TDS transfer line temperature, desorption time and cryodesorption time) as elucidated by the results of the screening design, at their central and high points, respectively, and in each case, the experiments were run in triplicate. We observed that the peak areas of the less volatile compounds (TDCIPP, TBOEP, TPHP and EHDPP) reduced at high levels of insignificant parameters. This could be attributed to the partial thermal decomposition that these compounds may undergo at higher desorption and cryodesorption temperatures^7^. By performing a paired samples *t* – test, it was revealed that the difference between the peak areas obtained at both the central and high points for the insignificant factors was not significant (*p* > 0.05). We therefore, used the central points for the insignificantly influential factors (to minimize thermal decomposition of the less volatile analytes), together with the modified optimal conditions of the significantly influential factors to test for the thermal extraction efficiency and validate the method.

The experimentally obtained peak area results when the insignificant factors were set are the central points are presented in Table 3. By using the predicted mean peak areas, prediction 95% confidence intervals were constructed and presented in Table 3. The experimentally obtained/validated mean peak areas (Table 3) generally fell with these confidence limits for TEP, TPP, TNBP, TCIPP and TPHP. For the rest of the compounds, the experimentally determined mean peak areas fell slightly below the lower limits of the 95% confidence intervals. From the results of a *t* – test, there was no significant difference (*p* > 0.05) between these mean peak values and their corresponding 95% confidence interval lower limits. This implies that the model predictions could be accepted for these compounds. Further, a scatter plot of the validated/experimentally obtained areas against the model predicted areas was generated and is presented in Fig. S3. The high value of the coefficient of determination (*r*^2^ = 0.9906) indicates a very strong agreement between the predicted and experimental results as well as the high accuracy of the proposed second order polynomial models. Moreover, the repeatability (CV%, Table 3) of the optimized procedure under the final modified optimal conditions was satisfactory, ranging from 6.7% for TCIPP to 12.3% for TBOEP.

The thermal desorption efficiency investigations were carried out by analyzing 3 separately spiked sorbent tubes, followed by desorbing the tubes for the second time. Desorption efficiency was calculated as the ratio of the peak area obtained on the first desorption to the total peak area obtained from the two desorptions and expressed as a percentage. The range of the desorption efficiency (%± CV) was from 99.8 ± 0.02% for TPHP to 100.0 ± 0.05% for TPP. Notably, these results showed no signs of carryover for all the target compounds.

Using the optimized conditions, we tested the performance of the method by evaluating appropriate validation parameters. The coefficient of determination (*r*^2^) values obtained ranged from 0.9942 for TEHP to 0.9998 for TEP, indicating that the responses were highly linear in the concentration range that was evaluated for all compounds. The values obtained were acceptable and in agreement with those obtained in the previous studies in which TE was investigated for flame retardants^7,8^. In addition, the linearity of the calibration curves was tested using Pearson correlation coefficient at a probability of 99%. In all cases, this test was passed, i.e., all the linearity coefficients were significant with 99% confidence. We also evaluated the relative response factor (RFF) CV values all of which were less than 10% for all calibration data points (Table 4, column 8).

Regarding the sensitivity of the method, the calculated instrumental LODs and LOQs ranged from 2.7 to 12.0 and 9.0 to 44.0 pg, respectively. These values correspond to LODs of 0.11 to 0.50 ng m^−3^ and LOQ of 0.38 and 1.83 ng m^−3^, respectively basing on the sampling volume of 0.024 m^3^. It is noteworthy to mention that our method is characterized by at least 6 times lower LODs than those obtained by Aragón *et al*.^5^. However, they are comparable with those obtained by Ramírez *et al*.^33^ but slightly lower than those obtained by Lazarov *et al*.^7^ probably due to differences in sample volumes. Table 4 summarizes the LOD and LOQ results obtained for all the target compounds. The results obtained demonstrated that the optimized method was effective in determining the target OPEs especially at low concentration levels.

The accuracy of the method was investigated at three calibration concentration levels: the lowest, intermediate and highest. Intra-day precision (n = 5) expressed in terms of coefficient of variation (CV) ranged from 1.5 to 9.9%, 2.2 to 13.2% and 0.7 to 9.8% at the lowest, intermediate and highest levels, respectively (Table 4). Generally, our results on intra-day precision of the method were in agreement with those obtained in some previous TE studies on SVOCs in which the CV values were less than 20%^5,7,8,33,34^. Moreover, the obtained CV values of less than 14%, indicated that the optimized procedure was characterized by good repeatability of the results. The recoveries of the target compounds ranged from 82% for EHDPP to 133% for TPHP, 90% for TEP to 115% for TDCIPP, and 96% for TEP to 124% for TBOEP, at the lowest, intermediate and highest calibration concentration levels, respectively. Generally the recovery range of our results fell with the range of 70 to 120% that is acceptable during the validation of analytical procedures depending on matrix complexity^35^. A few exceptions that were observed at the lowest and highest calibration concentration levels, were not significantly different (p > 0.05, student *t –* test) from the maximum acceptable limit (120%). These values affirm that the optimized method is accurate for the determination of the 10 OPEs in air samples. Furthermore, the obtained values indicated that the TE procedure was highly efficient to isolate gaseous OPEs present in the air sample matrix and hence applicable in the determination of these compounds in air samples collected from different environments. The results of inter-day (intermediate) precision (n = 5) CVs ranged from 4.4 to 11.8%, 2.5 to 12.5% and 3.2 to 12.5% at the lowest, intermediate and highest levels, respectively (Table 4). Our results agree with those obtained in some previous TE studies on SVOCs^8,33^ in which CVs less than 20% were obtained. The results indicated that the optimized procedure was characterized by high reproducibility, i.e., the results obtained were not sensitive to changes or variations in experimental times. It is worth noting that the precision values obtained for all the target compounds were less than 20% that is usually the acceptable limit for analytical method validation^36^.

With regard to the selectivity of the method, we compared the chromatograms obtained for the field blank, the intermediate calibration level and the real air sample (car) as shown in Fig. S1. There were no peak interferences observed at the retention times of the analytes in the SIM mode in the field blank except for TCEP. Some other peaks that were observed in the field blank tube are attributed to the internal standards and thermal decomposition which could result into the formation of other fragments^11^, or any other physical phenomena. These findings suggested that the spectrometric conditions that we used^8^, accorded high selectivity to the method for the target analytes. At all levels, all blank detections were less than the LOQs exept for TCEP, of which appropriate blank corrections were performed during the treatment of data obtained from real air sampling. Although some traces of the internal standards could be observed in the post conditioning analyses, perhaps due to some cold spots in the TDS, the percentage was as low as 4.3% and 2.6% for *d*_27_-TNBP and *d*_15_-TPHP, respectively, compared to the results obtained from the spiked tubes.

To test for the ruggedness of the method, we used the second order polynomial model equation for EHDPP:

1 $${\rm{Total}}\,{\rm{Peak}}\,{\rm{Area}}=-\,1.93033{\rm{E}}7+45358.8A+21252.6B+135283C-224.649{A}^{2}+155.24AB-12.6136AC-39.0508{B}^{2}-71.2049BC-229.315{C}^{2}$$

The aim was to study the effect of changing the cryofocusing temperature by 10% past the modified optimum, on the peak area of EHDPP. Substituting for A = 95 mL min^−1^ and C = 295 °C, equation 2 becomes;

2 $${\rm{Total}}\,{\rm{Peak}}\,{\rm{Area}}=-\,39.0508{B}^{2}+14994.955B+2577479.76$$

For simplicity, Equation 3 can be written as:

3 $$y=-\,39.0508{x}^{2}+14994.955x+2577479.76$$

From the Calculus of small changes,

4 $${\rm{\Delta }}y\approx \frac{dy}{dx}{\rm{\Delta }}x,\,{\rm{\Delta }}x\to 0$$

In this case,

5 $$x=20\,^\circ {\rm{C}},\,{\rm{\Delta }}x=2\,^\circ {\rm{C}},\,\frac{dy}{dx}=-\,78.1016x+14994.955$$

$\therefore {\rm{\Delta }}y=(\,-\,78.1016x+14994.955)\times 2.$ And when *x* = 20 °C, *y* = 2861758.54 Δ*y* = 26865.846 which corresponds to a 1% increase in peak area. This result shows that the method is not sensitive to random variations in the experimental conditions. It is therefore, highly rugged.

Concerning the breakthrough tests, after analyzing the two tubes for each test cycle, the concentration, in duplicate of each compound, obtained from the inlet tube was expressed as a percentage of the corresponding concentration obtained by analyzing tubes spiked with 1000 pg of the standard solution. The relative percentage recovery of all the compounds was greater than 95% up to when a volume of 0.024 m^3^ was passed through the tubes that were being tested. When 0.048 m^3^ was pumped through the tubes, the recovery of the compounds ranged from 50% for TEP to 90% for TNBP. We therefore considered 0.024 m^3^ as the appropriate sampling volume in real sampling applications. Moreover, breakthrough is considered to have occurred when the recovery in the inlet tube is less than 95%^9^.

Our results were compared with other studies as presented in Table 6. The optimum value for desorption flow in our study was 95 mL min^−1^ and is comparable with 100 mL min^−1^ which was used in the extraction of personal care products^33^ but which was used based on the supplier’s recommendation. Besides, our result was different from those used by Aragón *et al*.^5^ and Matsiko *et al*.^8^ because in these studies, univariate optimization was used. In Lazarov *et al*.^7^, no information is available on how the flow of 20 mL min^−1^ was selected. Our final desorption temperature value of 290 °C was comparable with those that were used in previous studies^5,7,8,33^, although in these studies, these values were selected based on the maximum temperature that is recommended for the sorbents that were used for extraction except for 300 °C which was the optimized value obtained by Matsiko *et al*.^8^. The final desorption time that we used in this study was comparable with those optimized in previous studies, whether optimized^5,8^ or selected^7,33^. Information on the TDS transfer temperature was not provided in some studies^5,7,33^, but the final value that we used in this study is comparable with the one optimized by Matsiko *et al*.^8^. For cryofocusing temperature, the modified optimum value in our study was different from the values used in the previous studies because the used values were either selected^5,7,33^ based on previous studies or arrived at by univariate optimization^8^. The optimum value for cryodesorption temperature in this study was different from those used in other studies due to the fact in some studies, this value was selected based on the maximum allowable value for the trap sorbent^5,33^ or univariate optimization was used^7,8^. The same reason applies to the difference in cryodesorption hold time. The detection and quantification limits in ng m^−3^ were calculated by dividing corresponding values in pg by the sampling volume (0.024 m^3^) and were comparable with those obtained in the previous studies^5,7,33^ on TE for determining SVOCs in air samples (Table 6). It is worth noting that the MQLs obtained in this study are also comparable with the results obtained in some studies in which solid phase extraction (SPE) was used for indoor active sampling of OPEs^37–39^. This indicates that the sensitivity of our method is satisfactory for the determination of gaseous phase OPEs in the indoor environments even at low concentration levels.

**Table 6:** Comparison with other TE studies on SVOCs.

Sorbent	Optimization	Analytes	Flow	DTp	DTm	TDsTr	CryoT	CryoDT	CDTm	MQL (ng/m^3^)	Reference
Tenax TA	Univariate	OPEs/PEs	50	320	10		0	320	5	7.0–670.0	^5^
PDMS/Tenax TA	Univariate	FRs	20	300	10	—	10	400	10	0.02–0.7	^7^
PDMS	Univariate	OPEs	76	320	10	320	−40	320	2	0.1–0.8^a^	^8^
Tenax TA	Univariate	PCPs	100	320	15	—	0	320	10	0.1–16.7	^33^
Tenax TA	Multivariate	OPEs	95	290	10	290	20	295	6	0.07–1.8	This Study

DTp; Desorption Temperature, DTm; Desorption Time, TDsTr; Thermal desorption system transfer line temperature, CryoT; Cryofocusing Temperature, CryoDT; Cryo – desorption Temperature, PEs; Phthalate esters, PCPs; Personal care products, FRs; Flame retardants.

^a^Units in pg/m^3^.

In conclusion, this work demonstrated the applicability of multivariate modelling in optimizing Tenax TA – thermal extraction for the determination of 10 OPEs in the gas phase fraction of air samples. With this optimization approach we were able to investigate a wider experimental domain, i.e., effect of the main factors, linear interactions and quadratic effects of the experimental factors on the magnitude of the response factors. Additionally, this optimization approach coupled with thermal extraction have proved to be time saving, and the amount of other resources such as the standard reagents, liquid nitrogen and helium are tremendously reduced. Without doubt, the versatility of multivariate optimization coupled with thermal extraction renders it a fast and cheap alternative to the commonly used univariate optimization and other multi-step extraction procedures such as accelerated solvent extraction. The relevance of the method was tested by collecting air samples from six indoor environments and TCEP was quantified in all the samples. Although this method is characterized by low method detection limits (0.02–0.5 ng m^−3^), the values are higher than those obtained in our previous study^8^, probably due to different sampling volumes. Noteworthy to mention, this method is characterized by low sampling volumes which may limit the detection of some target analytes, especially those that mainly partition in the particulate phase, in cases where the concentrations are at trace levels. Notwithstanding, the applicability multivariate optimization approach followed by TE in multi-residue analysis of SVOCs will be of interest.

## Materials and Methods

### Reagents and chemical standards

Acetone (pesticide grade) was obtained from Fisher Scientific, Fair Lawn, NJ, USA. Toluene (99.7% pure) was purchased from J.T. Baker, USA. Details of the chemical and internal standard solutions of the target OPEs have been provided in our previous study^8^. Stock solutions were prepared in toluene and appropriate dilutions were made with acetone. Additionally, the physical properties of the target compounds are presented in Table S4.

### Sampling cartridges

Standard glass sorbent cartridges (180 mm, length 6 mm, outer diameter and 4 mm inner diameter, Gerstel), each packed with approximately 60 mm/approximately 180 mg of Tenax TA adsorbent, were used for adsorption/sampling and then thermal extraction, prior to chromatographic analysis. Tenax TA is a polymeric material based on 2, 6-diphenyl–*p*–phenylene oxide that is used as an adsorbent material in various analytical applications. Its physical properties with regard to topography, crystal structure and thermal stability were evaluated by Alfeeli *et al*.^40^. Before use, the sorbent packed tubes were thermally cleaned in a Gerstel Tube conditioner for eight hours at a temperature of 320 °C and under nitrogen gas flowing at 75 mL min^−1^. Subsequent pre-cleaning was carried out for 2 hours at the same conditions of temperature and gas flow rate. Thereafter, the clean tubes were placed in storage tubes which were then capped with long-term storage plastic caps combined with PTFE ferrules and then stored in airtight sealable plastic jars, which were then kept under a nitrogen atmosphere to prevent contamination from the immediate surroundings.

### Tube loading and desorption

To optimize, calibrate and validate the method for the TE procedure under the current study, it was necessary to spike the sorbent tubes with standard solutions. For all the calibration concentration levels used at each stage, 1 μL of the standard solution in acetone was spiked onto the sorbent tube using a 10 μL GC/MS auto sampler syringe and thereafter, purged with 99.99% nitrogen gas at a flow rate of 75 mL min^−1^ for 2 minutes. The liquid mixture vapourized in this gas flow constitution and the analytes were adsorbed onto the sorbent in vapour phase. Purging was carried out to ensure that the optimization and validation procedures were consistent with real air sampling. Acetone was chosen as the solvent because of its weak retention characteristics on Tenax TA^40^. Notably, 2 minutes of nitrogen gas flow (150 mL) was adequate to dry the solvent while making sure that the analytes of interest were not expelled out of the tube. The spiked tubes were then placed in storage tubes which were capped with long-term storage plastic caps combined with PTFE ferrules, wrapped in aluminium foil and stored at −20 °C for about 4 hours for the sorbent bed to equilibrate, prior to extraction. A standard two stage desorption strategy was used for analysis.

### Experimental Design/Statistical Treatment

All experiments in the screening and refining/modelling procedures were designed using Statgraphics Centurion 18 (StatPoint Technologies, Inc) software. The goal of the designs used in this study was to maximize the response factors (compound specific chromatographic peak areas). Other statistical analyses were performed by Microsoft excel, IBM^®^ SPSS^®^ Statistics Version 21 and Origin^®^ Pro Version 9.1.

### Screening

We planned screening experiments using the eighth fractional factorial design (2^*K*−3^, K = 7) with 4 center points to determine which factors affected the compounds’ peak areas significantly. The seven factors that were investigated, together with their ranges are as follows; desorption flow rate (20–100 mL min^−1^), desorption temperature (260–320 °C), thermal desorption system (TDS) transfer line temperature (260–320 °C), desorption hold time (5–15 min), cryofocusing temperature (−100–+60 °C), cryodesorption temperature (260–320 °C) and cryodesorption hold time (2–10 min). These factors and their ranges were considered basing on preliminary experiments, previous studies^5–8,21–23^, boiling points of the analytes (Table S4), and the thermal stability of Tenax TA^40^.

A second order polynomial model was selected in which all the main factor effects and the quadratic effect of desorption flow rate were automatically included in the design. By using the forward algorithm, 19 desired runs were selected, i.e., 16 runs at the factorial points and 3 runs at the center of the design. The central point was repeated twice (six replicates in total) to ensure that the repeatability was within the normal dispersion range. The experiments were ran in a random manner in order to minimize the effect of uncontrolled variables^25^. These experiments were carried out in duplicate by spiking 1 μL of the standard solution containing 1000 pg of each target compound on separate sorbent tubes.

### Modelling and Optimization

The significantly influential factors as revealed by the results of the screening experiments, were used to develop second order polynomial models for evaluating the response factors. The experiments were planned using the Box – Behnken design (BBD) with 15 experimental runs (12 at factorial points and 3 at the center). The significance level for each experimental factor in the response factor mathematical models was evaluated by the analysis of variance (ANOVA). These experiments were carried out in duplicate by spiking 1 μL of 2000 pg standard solution on separate sorbent tubes. The central point was also repeated twice to ensure that repeatability was within the normal dispersion range. In the selected second order models (equation 6), the main factors, linear interaction and quadratic effects were evaluated.

6 $$Y={\beta }_{0}+{\beta }_{1}A+{\beta }_{2}B+{\beta }_{3}C+{\beta }_{11}{A}^{2}+{\beta }_{12}AB+{\beta }_{13}AC+{\beta }_{22}{B}^{2}+{\beta }_{23}BC+{\beta }_{33}{C}^{2}+\varepsilon $$

where, Y is the single response (compound specific peak area) to be modelled, β is the regression coefficient, A, B and C are the experimental factors and ε is the experimental error. Subsequently, the models, together with Derringer’s desirability function, were used to determine the optimal conditions for the TE procedure under the current study.

### Method validation and Quality Assurance/Control (QA/QC)

To test for the linearity of the method, the tubes were spiked separately in triplicate with 1 μL of each of the calibration concentration solutions (50–2000 pg) containing 100 pg of the internal standards^9^. Calibration was then done by plotting the area ratio, $({A}_{i}/{A}_{sur})$ (where *A*_*i*_ is the peak area of the target analyte and *A*_*sur*_ is the peak area of the surrogate standard) against the concentration of the target analyte. Due to the unavailability of certified reference materials, the accuracy of the method in terms of recovery, method repeatability and intermediate precision was tested by spiking five tubes separately with the lowest, intermediate and highest concentration levels. Repeatability was tested by performing the experiments on the same day, while intermediate precision was tested by performing the experiments for five consecutive days, one experiment per day for each of the three calibration levels. In order to ascertain whether the target compounds could be present in the air samples, we examined the limits of detection and quantitation (LOD and LOQ). These were calculated using the formulae; 3*σ* and 10*σ* where *σ* is the standard deviation of the analytical results obtained by analyzing 5 desorption tubes spiked with the lowest calibration concentration.

QA/QC was ensured at different levels. Post conditioning analyses were carried out by analysing six pre-cleaned tubes without spiking them with the internal standards. We also investigated post conditioning analyses when the tubes were spiked with the internal standards. Laboratory and field blanks were carried out by connecting six tubes separately to the pump and then exposing them to the laboratory air and an office environment air, respectively, in the passive diffusive mode (without starting the pump) for one minute^34^. They were then capped, wrapped in a clean aluminuim foil and kept at −20 °C until analysis. Breakthrough volume tests were carried out by connecting two pre-cleaned sorbent tubes in series with a silicone tube, with the inlet tube spiked with 1000 pg of the standard solution. Volumes of clean laboratory air ranging from 0.003 to 0.048 m^3^ were pumped through the tubes at a rate of 200 mL min^−1^. At the end of each experiment, the two tubes were analysed separately. For each volume, the experiments were run in duplicate.

### Sampling

The air samples were collected based on active air sampling with a variable flow air pump (QC-IC portable membrane pump, 50–500 mL min^−1^, Beijing Labour Protection Science Research Institute, CN). Air samples were pumped through the preconditioned sorbent tubes at a flow rate of 200 mL min^−1^ for 2 hours with a total volume of 0.024 m^3^ of air collected. Prior to sampling, we calibrated two pumps using a soap film flowmeter (0101–0113 (1–10–100 mL)) as illustrated in Fig. S4A. The inlet port of the pump was connected to the Tenax TA packed Gerstel tube via a silicone delivery tube. A quartz fiber filter (QFF) was used to seal the sampling end to prevent air borne particles from entering the tube. The values obtained for the two tested pumps (n = 3, for each set rate) were plotted and fitted with exponential decay (order 1) functions as shown in Fig. S4B. By inspection, it can be seen that pump A gave the best calibration results. It is worth noting that, calibration was carried out before and after each sampling session to ensure consistency in the sampled volume. The pump was positioned on a stand, 0.8 m above the floor level in each of the studied indoor environments.

### Instrumental analysis

Thermal desorption was performed using a commercial desorption unit, TDS-3 (Gerstel) connected to a programmed-temperature vapouriser (PTV) injector/cooled injection system (CIS – 3) (Gerstel) by a heated transfer line. Other analysis details are provided in Text S1.

## Supplementary information

Supplementary Information for; Multivariate Optimization of Tenax TA-Thermal Extraction for Determining Gaseous Phase Organophosphate Esters in Air Samples

## Data Availability

The data associated with this manuscript has been availed as far as possible.
