# Supplementary material for: Multivariate Optimization of Tenax TA-Thermal Extraction for Determining Gaseous Phase Organophosphate Esters in Air Samples
Source: Sci Rep. 2019 Mar 4;9:3330. doi: 10.1038/s41598-019-40119-2 (PMC6399288; doi:10.1038/s41598-019-40119-2)
Supplement: Supplementary file 1 — Supplementary Information for; Multivariate Optimization of Tenax TA-Thermal Extraction for Determining Gaseous Phase Organophosphate Esters in Air Samples [file 41598_2019_40119_MOESM1_ESM.pdf]

Supplementary Information for;

Multivariate Optimization of Tenax TA-Thermal Extraction for Determining Gaseous Phase Organophosphate Esters in Air Samples

Julius Matsiko,[1,2] Honghua Li,[1] Pu Wang,[1] Huizhong Sun,[1,2] Shucheng Zheng,[1,2] Dou Wang,[1,2] Weiwei Zhang,[1,2] Yanfen Hao,[1,2] Peijie Zuo,[1,2] Yingming Li,[1] Qinghua Zhang,[1,2,3,]* Jianqing Zhang[4,]* and Guibin Jiang [1,2]

[1]State Key Laboratory of Environmental Chemistry and Ecotoxicology, Research Center for Eco-Environmental Sciences, Chinese Academy of Sciences, Beijing 100085, China

[2]University of Chinese Academy of Sciences, Beijing 100049, China

[3]Institute of Environment and Health, Jianghan University, Wuhan 430056, China

[4]Shenzhen Center for Disease Control and Prevention, Shenzhen 518055, China

Corresponding author

*****qhzhang@rcees.ac.cn, Tel: +86 10 6284 9818 (Q. Zhang)

*****969676617@qq.com, Tel: +86 755-25531955 (J. Zhang)

Text S1

Instrumental analysis

Thermal desorption was performed using a commercial desorption unit, TDS-3 (Gerstel) connected to a programmed-temperature vapouriser (PTV) injector/cooled injection system (CIS – 3) (Gerstel) by a heated transfer line. The CIS was equipped with a baffled glass liner and was cooled by liquid nitrogen for analyte trapping prior to injection into a chromatographic column for analytical separation and detection. Moreover, a baffled glass liner has been shown to provide good responses in a number of studies involving SVOCs.[1,2,3] The temperature program for desorption was 30 ℃ (delay time of 0.2 minutes), then ramped at 130°C/minute to 290°C, held for 10 minutes. The desorption unit, TDS-3 was operated in a splitless mode. The temperature of the transfer line was set at 290°C. The CIS was cooled with liquid nitrogen to 20°C. After desorption and cryotrapping, cryo-desorption was carried out at 10°C per second from 20°C to 295°C ,held for 6 minutes, for quantitative sample transfer into the chromatographic column for analyte separation. Meanwhile, the desorbed analytes were being carried in helium gas at $95 \mathrm{mLmin}^{-1}$. The CIS was also operated in a splitless mode. The TDS-3 device was connected to a Shimadizu GC coupled to a MS (GCMS-QP2010 Ultra). Gas chromatography/mass spectrometry parameters have been reported in our previous study.[1]

Experimental Factors and their letter codes	Experimental Factor Levels and their codes		
	Low (-1)	Center (0)	High (+1)
Desorption Flow (A)	20	60	100
Desorption Temperature (B)	260	290	320
TDS Transfer Temperature (C)	260	290	320
Desorption Time (D)	5	10	15
Cryofocusing Temperature (E)	-100	-20	60
Cryodesorption Temperature (F)	260	290	320
Cryodesorption Time (G)	2	6	10

	Experimental factor settings at each run							Response Factors (Compound Specific Chromatographic Areas)									
Run	A	B	C	D	E	F	G	TEP	TPP	TNBP	TCEP	TCIPP	TDCIPP	TBOEP	TPHP	EHDPP	TEHP
1	100	320	320	5	60	260	2	451704.5	1056466.0	675385.5	133531.5	88479.5	45542.0	28719.5	253084.5	453589.0	1211914.5
2	100	260	260	15	60	320	2	526735.5	1286762.0	830984.0	178161.0	109761.0	63169.0	61049.0	352863.5	621392.5	1719607.0
3	20	260	320	15	60	260	2	408225.0	1384418.0	1135416.0	244902.0	145930.0	93538.0	61170.0	499499.0	930945.0	2315544.0
4	20	260	320	5	60	320	10	640922.5	1687884.0	1129207.5	220883.5	144792.5	79661.5	55578.5	425920.0	696135.5	2046014.0
5	100	260	320	15	-100	260	10	723155.5	1881437.5	1251537.0	242739.5	158709.5	90071.0	69104.5	439617.5	927317.5	2632893.0
6[a]	60	290	290	10	-20	290	6.	308746.5	1324942.5	926822.5	266503.5	124268.5	122458.0	139601.0	626554.5	1313301.0	3161711.0
7	60	290	290	5	-100	260	10	226337,0	1307480.5	1028516.0	288552.5	148598.0	98278.5	69579.0	526802.5	962212.5	2492056.5
8[a]	60	290	290	10	-20	290	6	289871,0	1455719.0	802766.0	282503.5	112137.0	106589.0	140179.0	560532.0	1001512.5	2744051.5
9	100	260	320	5	-100	320	2	384496,0	1025152.0	650979.5	56484.0	75923.5	11046.5	4290.5	57860.5	103947.0	378548.5
10	20	320	260	5	60	320	2	385844.5	1115201.0	774053.0	79814.5	89805.5	13996.5	4086.5	70800.5	136561.0	846249.5
11[a]	60	290	290	10	-20	290	6	263023,0	1144365.0	694889.0	287697.0	112258.0	111610.0	123315.0	490482.0	991590.0	2412535.0
12	20	260	260	5.0	-100	260	2	539502.5	1462634.0	910382.0	128325.5	103434.0	26100.5	9539.0	142639.5	314977.5	1315715.5
13	100	320	260	15	-100	260	2	316586.5	1407336.5	1058002.0	289870.0	146214.5	111634.0	84859.5	563080.5	1125788.0	2908618.5
14	20	260	260	15	-100	320	10	255369.5	1198366.5	705135.5	12251.0	47336.0	3121.0	1669.0	17755.0	24012.5	98961.0
15	20	320	260	15	60	260	10	309795,0	851746.0	524690.0	28150.0	62033.0	6275.0	779.0	20107.0	39216.0	437800.0
16	20	320	320	15	-100	320	2	300141.5	1365388.5	942845.0	300560.0	134072.5	121387.5	93982.5	597146.5	1141368.0	2759217.5
17	100	320	260	5	-100	320	10	793397,0	2077767.5	1455719.5	328124.0	187987.5	134800.5	114968.0	754426.5	1463732.0	3619735.0
18	100	260	260	5	60	260	10	408328.5	1401793.0	990983.0	209843.0	123675.0	82130.0	61808.5	448626.0	802767.5	2182119.5
19	100	320	320	15	60	320	10	272184,0	1121369.5	928838.0	236523.5	131648.0	86856.5	53871.5	441180.0	856743.0	2071634.0

[a] refers to the center points of the screening design.

Table S1. Experimental factors, codes, and levels in the 2^{7-3} eighth fractional factorial design and the corresponding average compound peak areas.

Experimental Factors and Letter Codes	Experimental Factor Levels and their codes		
	Low (-1)	Center (0)	High (+1)
Desorption Flow (A)	20	60	100
Cryofocusing Temperature (B)	-100	-20	60
Cryodesorption Temperature (C)	260	290	320

Experimental factor settings at each run

Run	A	B	C	Response Factors (Compound Specific Chromatographic Areas) TEP		TPP		TNBP		TCEP		TCIPP	
				Actual	Predicted	Actual	Predicted	Actual	Predicted	Actual	Predicted	Actual	Predicted
1	20.0	-20.0	320.0	922208.0	1170880.0	2341327.0	3001260.0	1516043.5	1917380.0	312759.5	374208.0	159056.5	197382.0
2	60.0	-100.0	260.0	857785.0	877999.0	2238820.0	2621740.0	1414727.5	1722920.0	237028.5	258081.0	160049.0	190166.0
3	20.0	-100.0	290.0	710402.0	615658.0	2474229.5	2230460.0	1761650.5	1607190.0	294577.0	251074.0	193083.5	184836.0
4	100.0	-20.0	320.0	1695451.5	1620920.0	4424775.5	4563920.0	2803856.5	2957600.0	590934.0	568484.0	290491.0	312360.0
5[a]	60.0	-20.0	290.0	1507348.0	1606140.0	4047543.5	4484590.0	2580844.5	2936570.0	498191.0	548977.0	272580.0	314910.0
6[a]	60.0	-20.0	290.0	1588502.5	1606140.0	4340267.5	4484590.0	2805147.0	2936570.0	519892.5	548977.0	291682.0	314910.0
7	100.0	-20.0	260.0	1629748.5	1381080.0	4598243.0	3938310.0	2980115.0	2578780.0	613775.5	552327.0	328037.5	289713.0
8	20.0	60.0	290.0	617384.0	388925.0	3503504.5	3226480.0	2353480.5	2260340.0	478381.0	437985.0	252880.0	244672.0
9	60.0	60.0	320.0	481635.5	461421.0	4113335.5	3730420.0	2992992.5	2684800.0	671822.5	650770.0	321764.5	291648.0
10	60.0	60.0	260.0	236390.5	390320.0	3567739.0	3983910.0	2731648.5	2978530.0	684918.5	702864.0	328521.5	358599.0
11	100.0	-100.0	290.0	941389.0	1169850.0	2952669.0	3229690.0	1955490.5	2048630.0	207962.0	248358.0	209483.5	217692.0
12	60.0	-100.0	320.0	1114979.5	961050.0	3466242.5	3050070.0	2352306.0	2105430.0	353064.0	335118.0	269365.5	239288.0
13	100.0	60.0	290.0	314529.5	409273.0	4032421.5	4276190.0	3075998.5	3230460.0	778378.5	821881.0	370401.5	378649.0
14	20.0	-20.0	260.0	1182047.0	1256580.0	3591177.0	3452030.0	2361172.0	2207430.0	342973.0	365423.0	259728.0	237859.0
15[a]	60.0	-20.0	290.0	1722575.0	1606140.0	5065956.0	4484590.0	3423728.0	2936570.0	628848.0	548977.0	380469.0	314910.0

Run	A	B	C	TDCIPP		TBOEP		TPHP		EHDPP		TEHP	
				Actual	Predicted	Actual	Predicted	Actual	Predicted	Actual	Predicted	Actual	Predicted
1	20.0	-20.0	320.0	112062.5	136903.0	116688.0	109911.0	470025.0	564627.0	1057562.5	1195010.0	3310298.0	3938950.0
2	60.0	-100.0	260.0	38897.0	42543.6	18023.0	6039.5	153062.0	144445.0	463195.0	488782.0	2217466.0	2727000.0
3	20.0	-100.0	290.0	69213.5	58102.9	40123.5	31391.8	308220.0	297996.0	714784.0	626298.0	3327482.5	3121420.0
4	100.0	-20.0	320.0	235183.5	227719.0	221664.5	200949.0	999300.0	980459.0	2158696.0	2095800.0	6533647.0	6837110.0
5[a]	60.0	-20.0	290.0	198883.0	219836.0	157377.0	174225.0	812895.0	922826.0	1881157.5	2122790.0	6119793.0	6921490.0
6[a]	60.0	-20.0	290.0	205903.5	219836.0	159285,0	174225.0	823900.5	922826.0	1992677.0	2122790.0	6740108.0	6921490.0
7	100.0	-20.0	260.0	219622.5	194782.0	163279.5	170057.0	870684.5	776083.0	2086644.0	1949190.0	6206228.5	5577570.0
8	20.0	60.0	290.0	196239.5	175045.0	121144.0	115938.0	796777.5	693559.0	1581362.0	1469500.0	5166159.0	5047030.0
9	60.0	60.0	320.0	262223.5	258577.0	234485.5	246469.0	1029696.0	1038310.0	2527982.5	2502400.0	7560157.0	7050630.0
10	60.0	60.0	260.0	259307.0	273037.0	247585.5	232077.0	1009825.5	1094200.0	2618338.0	2667300.0	7450197.0	7872790.0
11	100.0	-100.0	290.0	50428.0	71622.3	15071.0	20277.3	254986.5	358205.0	451951.0	563815.0	3112110.0	3231230.0
12	60.0	-100.0	320.0	125466.0	111736.0	60761.5	76270.4	631771.5	547394.0	1056406.5	1007440.0	4840960.0	4418370.0
13	100.0	60.0	290.0	320904.0	332015.0	323235.5	331967.0	1393097.0	1403320.0	3305604.5	3394090.0	8877605.5	9083670.0
14	20.0	-20.0	260.0	107645.0	115109.0	35465.0	56180.2	403102.0	421943.0	924965.0	987865.0	4632754,0	4329290.0
15[a]	60.0	-20.0	290.0	254722.5	219836.0	206013.0	174225.0	1131682.0	922826.0	2494531.5	2122790.0	7904560,0	6921490.0

[a] refers to the center points of the screening design.

Table S2. Box-Behnken response surface design matrix and the corresponding average (actual and model predicted) compound peak areas

Table S3
Analysis of variance (ANOVA) of the response surface regression model for EHDPP.

Source	Sum of squares	Degrees of freedom	Mean square	F-ratio	p-value
A:Desorption flow	1.73373E12	1	1.73373E12	28.33	0.0031
B:Cryofocusing Temperature	6.74721E12	1	6.74721E12	110.25	0.0001
C:Cryodesorption Temperature	6.25705E10	1	6.25705E10	1.02	0.3584
AA	4.77031E11	1	4.77031E11	7.79	0.0384
AB	9.87117E11	1	9.87117E11	16.13	0.0102
AC	9.16439E8	1	9.16439E8	0.01	0.9074
BB	2.3063E11	1	2.3063E11	3.77	0.1099
BC	1.16816E11	1	1.16816E11	1.91	0.2256
CC	1.5727E11	1	1.5727E11	2.57	0.1698
Lack of fit	1.197E12	6	1.996E11	1.869	0.389
Pure error	2,135E11	2	1.068E11		
Total error	3.05997E11	5	6.11994E10		
Total (corr.)	1.07139E13	14			

R-squared = 97.1439 percent
R-squared (adjusted for d.f.) = 92.003 percent
Standard Error of Est. = 247385.
Mean absolute error = 112933.
Durbin-Watson statistic = 1.48657 (P=0.2925)
Lag 1 residual autocorrelation = 0.0000359516

Table S3. Analysis of variance (ANOVA) of the response surface regression model for EHDPP.

No.	Compound	Practical Abbreviation (PRAB)	B.p (℃)	Structure	CAS Number	MW	Log K_{ow}	Koc	Vapour Pressure (Pa)	$Log\ K_{OA}$
1	Triethyl phosphate	TEP	216	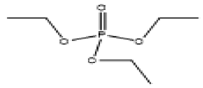	78-40-0	182.16	0.80	36	5.25E+01	6.1
2	Tri-n-propyl phosphate	TPP	254	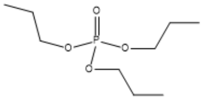	513-08-6	224.24	1.87	676	5.77E-01	6.4
3	Tri-n-butyl phosphate	TNBP	289	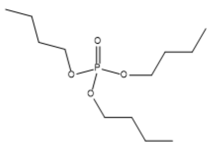	126-73-8	266.32	3.60	977	1.71E00	8.2
4	Tris(2-chloroethyl) phosphate	TCEP	351	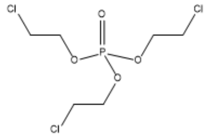	115-96-8	285.49	1.47	150	1.44E-02	7.4
5	Tris(2-chloroisopropyl)phosphate	TCIPP	342	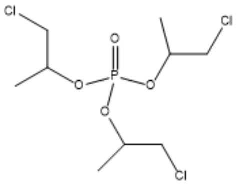	13674-84-5	327.56	2.59	275	2.69E-03	8.2
6	Tris(1,3-dichloro-2-propyl) phosphate	TDCIPP	457	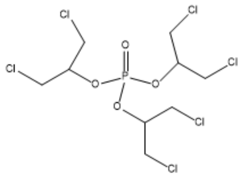	13674-87-8	430.90	3.27	1440	5.43E-06	10.6
7	Tris(2-butoxyethyl) phosphate	TBOEP	414	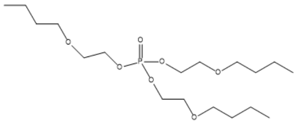	78-51-3	398.48	3.75	1020	3.33E-06	13.1
8	Triphenyl phosphate	TPHP	370	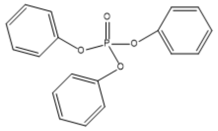	115-86-6	326.29	4.59	2630	8.37E-03	8.5
9	2-Ethylhexyl diphenyl phosphate	EHDPP	375	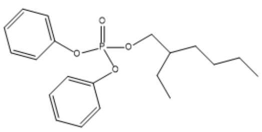	1241-94-7	362.40	5.73	9499	6.20E-04	11.3
10	Tris(2-ethylhexyl) phosphate	TEHP	220	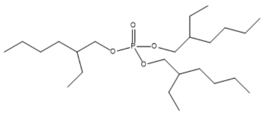	78-42-2	434.64	9.49	617000	1.10E-05	15.0

Table S4. Names, abbreviations, structures and other properties of the target compounds

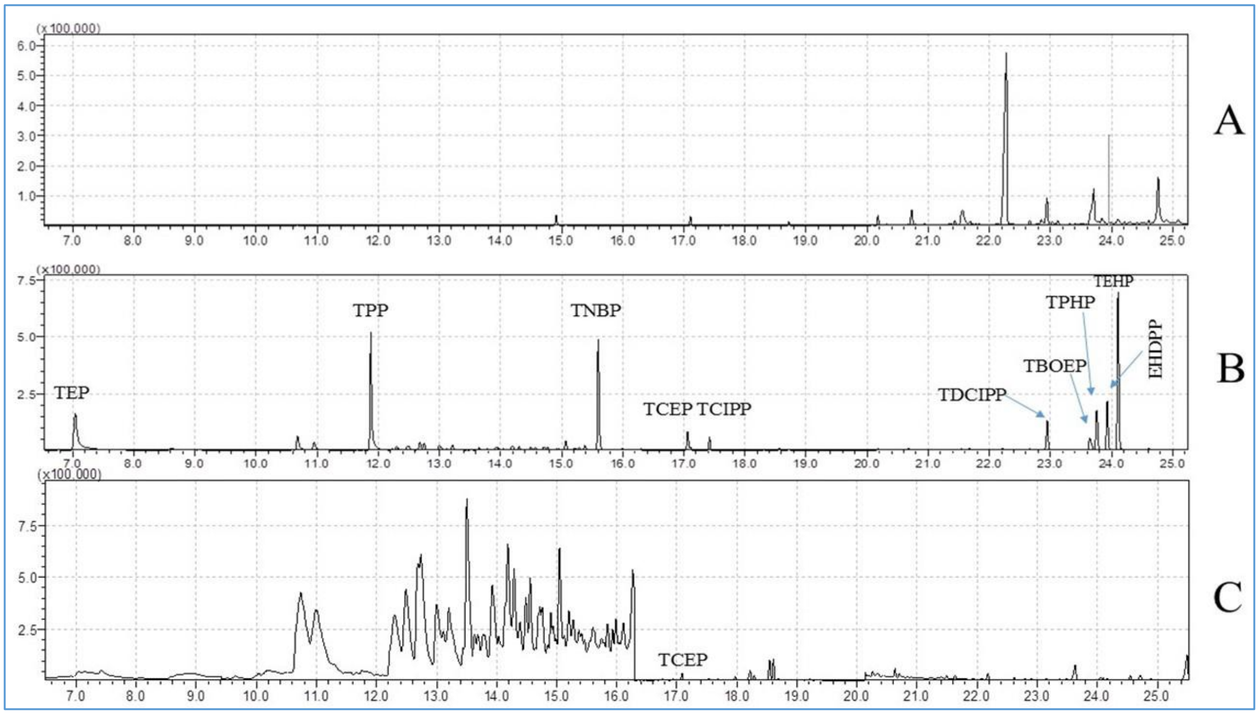

Figure S1. Total Ion Chromatograms (TIC) laboratory blank tube (A), intermediate calibration concentration level (B) and real air sample collected from the car (C)

A

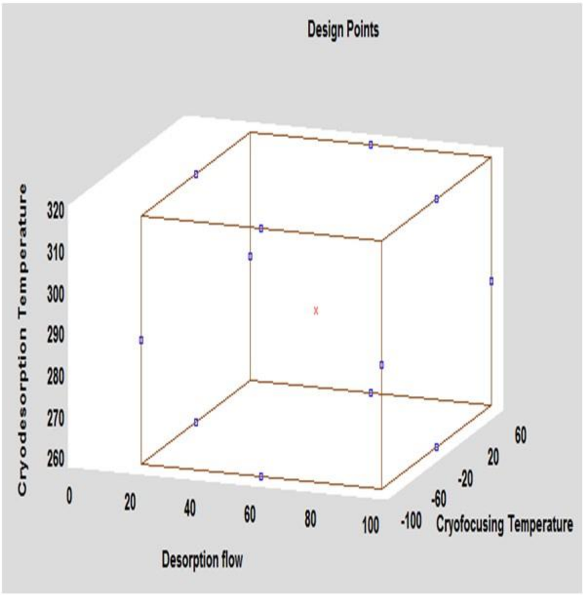

B

Run	A	B	C
1	-1	0	+1
2	0	-1	-1
3	-1	-1	0
4	+1	0	+1
5	0	0	0
6	0	0	0
7	+1	0	-1
8	-1	0	0
9	0	+1	+1
10	0	+1	-1
11	+1	-1	0
12	0	-1	+1
13	+1	+1	0
14	-1	0	-1
15	0	0	0

Figure S2. Box-Benken Design plot (A) and Matrix codes (B).

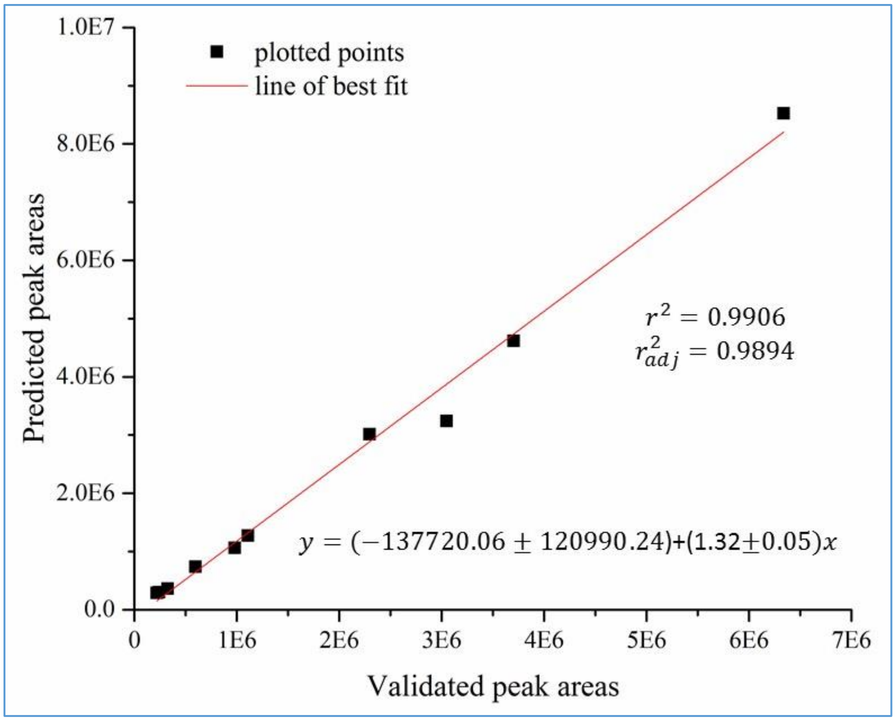

Figure S3. Scatter plot illustrating the relationship the modelled and experimental/validated peak areas.

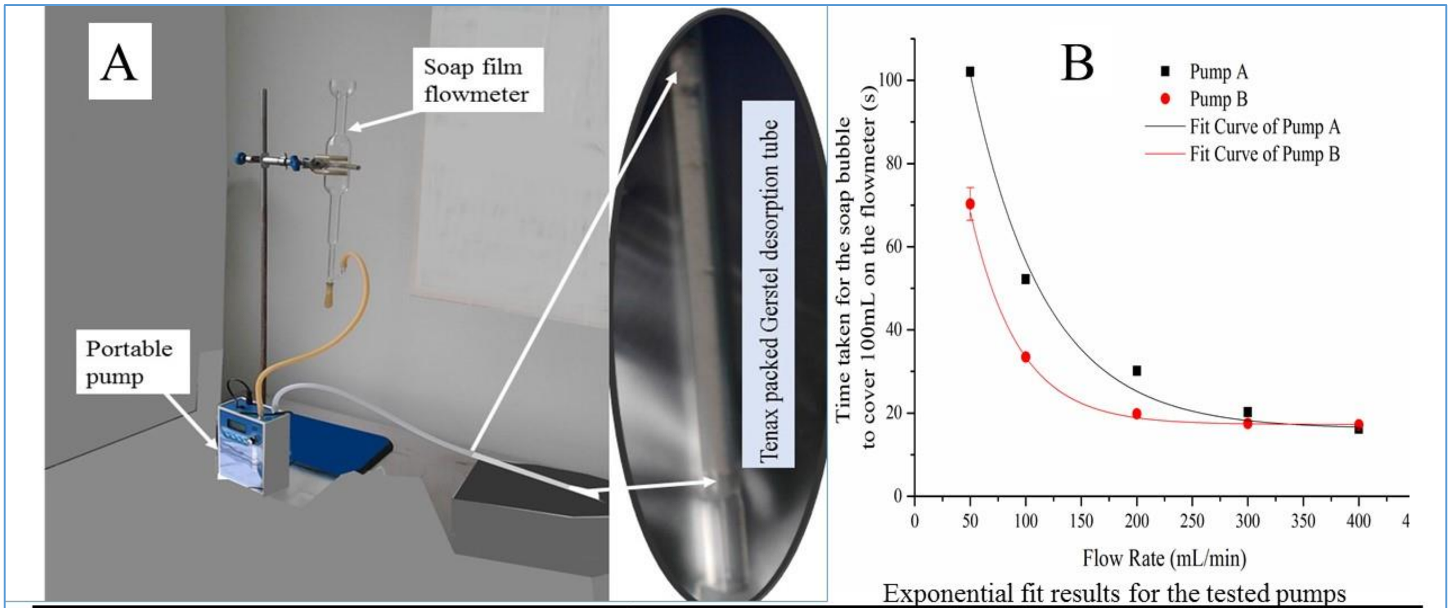

Exponential fit results for the tested pumps

**Equation**	$y = y_0 + A_1 e^{-x/t_1}$						**k**	**tau**		
	y_o		A_1		t_1		$1/t_1$	$t_1 \ln 2$		
									χ^2_{red}	r^2_{adj}
	value	SE	value	SE	value	SE	Value			
**Pump A**	16.2	1.1	180.1	25.5	66.9	11.8	0.01	46.4	411.8	0.9928
**Pump B**	17.3	0.2	161.9	24.4	43.4	2.9	0.02	30.1	1.1	0.9996

[a]k and [b]tau are derived constants

Figure S4. Calibration set up (A) and calibration curves (B, n=3) for the two tested QC-IC pumps.

References

1. Matsiko, J. et al. Stir bar sorptive extraction and thermal desorption - gas chromatography/mass spectrometry for determining phosphorus flame retardants in air samples. *Anal. Methods* **10**, 1918-1927 (2018).

2. León, V.M., Álvarez, B., Cobollo, M.A., Muñoz, S. & Valor, I. Analysis of 35 priority semivolatile compounds in water by stir bar sorptive extraction–thermal desorption–gas chromatography–mass spectrometry: I. Method optimisation. *J. Chromatogr., A* **999**, 91-101 (2003).

3. Cacho, J.I., Campillo, N., Viñas, P. & Hernández-Córdoba, M. Stir bar sorptive extraction coupled to gas chromatography–mass spectrometry for the determination of bisphenols in canned beverages and filling liquids of canned vegetables. *J. Chromatogr., A* **1247**, 146-153 (2012).
